# Enhanced odontoblast regeneration and anti-inflammatory effects of nanocurcumin-loaded BM-MSC-derived exosomes in an LPS-induced periodontitis rat model

**DOI:** 10.1186/s12903-026-09697-2

**Published:** 2026-09-03

**Authors:** Nehad M. Abd-elmonsif, Ahmed Morsy, Mona El Deeb

**Affiliations:** 1 Department of Oral Biology, Faculty of Oral and Dental Medicine, Future University in Egypt, Cairo, Egypt; 2 Independent Researcher, New York, NY USA

**Keywords:** Periodontitis, Odontoblasts, Mesenchymal stem cell exosomes, Nanocurcumin, Interleukin-10, Regenerative dentistry

## Abstract

**Background:**

Periodontitis is commonly known as a chronic inflammatory disease which adversely affects pulpal and periodontal tissues, including odontoblasts. Bone marrow mesenchymal stem cell-derived exosomes (BM-MSC-Exo) and nanocurcumin (NCUR) possess anti-inflammatory and regenerative properties; however, their combined effects on odontoblasts remain insufficiently explored. The research target was to assess the histological, histochemical, and anti-inflammatory effects of BM-MSC-Exo and NCUR-loaded BM-MSC-Exo on odontoblasts of LPS-induced periodontitis in rats.

**Methods:**

Twenty-eight male albino rats were assigned at random into four groups (*n* = 7 each): control, periodontitis, periodontitis treated with BM-MSC-Exo, and periodontitis treated with NCUR-loaded BM-MSC-Exo. Periodontitis was induced via intra-periodontal LPS injections. Histological evaluation was performed using hematoxylin and eosin staining, and collagen synthesis was analyzed using Masson’s trichrome stain. Morphometric analysis of newly formed collagen and enzyme-linked immunosorbent assay (ELISA) of interleukin-10 (IL-10) levels were conducted.

**Results:**

Untreated periodontitis caused marked odontoblastic degeneration, reduced predentin thickness, and decreased collagen formation. Treatment with BM-MSC-Exo significantly improved odontoblastic architecture, enhanced collagen deposition, and increased IL-10 expression. NCUR-loaded BM-MSC-Exo demonstrated superior regenerative and anti-inflammatory effects, characterized by improved odontoblastic organization, increasing the area of newly formed collagen, and significantly elevated IL-10 levels compared with all other groups (*p* < 0.001).

**Conclusions:**

BM-MSC-Exo exerts regenerative effects on odontoblasts in periodontitis, which are further enhanced by NCUR loading. NCUR-loaded BM-MSC-Exo declares an encouraging cell-free medicinal strategy for regeneration of periodontal and pulpal tissues.

**Graphical abstract:**

**Figure:**
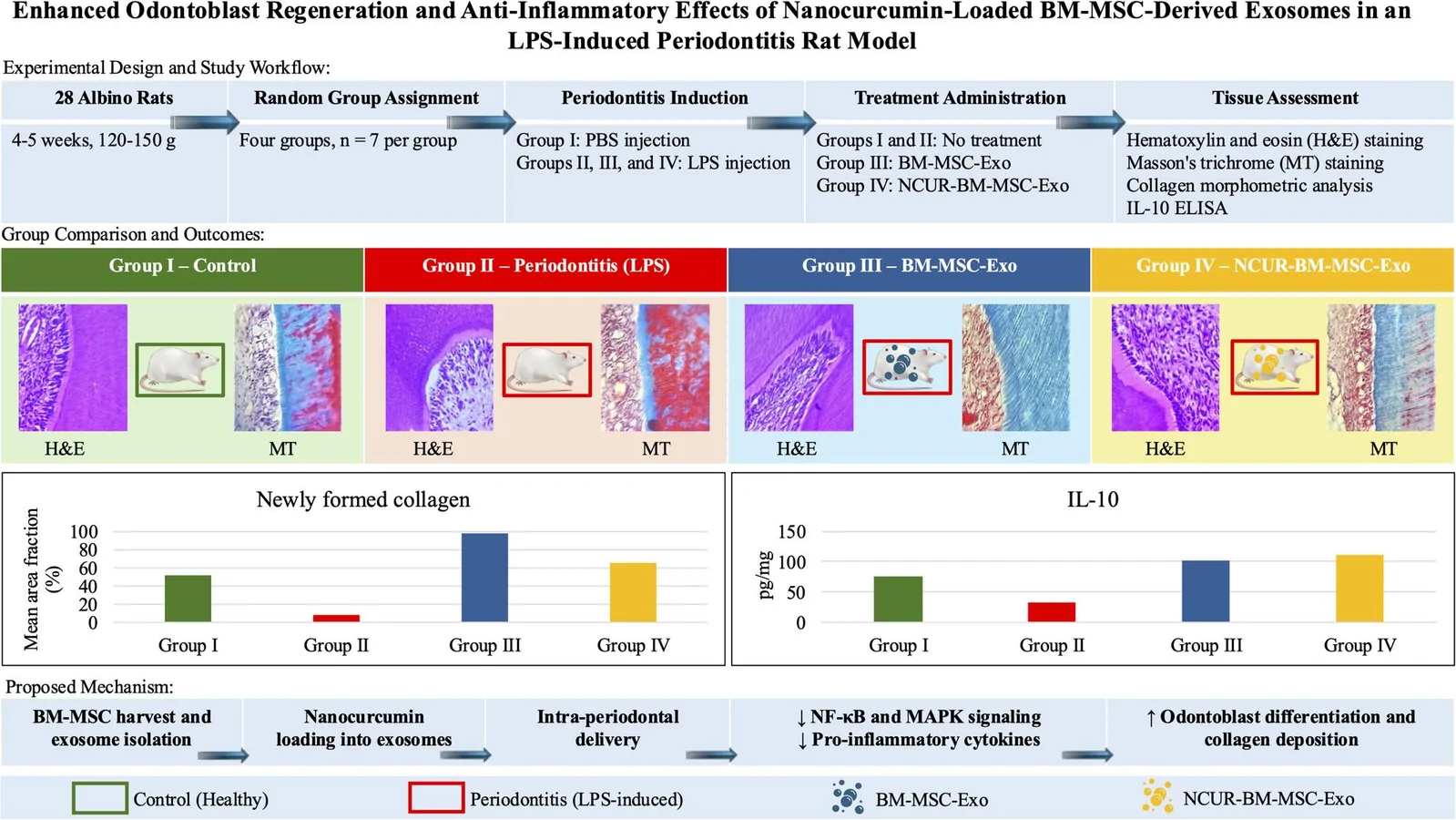

## Background

Odontoblasts are highly differentiated, non-dividing cells originating from the dental pulp, responsible for dentin formation and capable of functioning as sensory receptors [1, 2]. Periodontitis represents a widespread chronic inflammatory disease with significant public health implications, being strongly associated with tooth loss and systemic inflammation [3]. This chronic inflammatory condition is initiated by microbial dysbiosis, in which bacterial products, including lipopolysaccharides (LPS), trigger sustained host immune responses that ultimately drive destruction of periodontal tissue [4].

The apical foramen, auxiliary canals, and exposed dentinal tubules allow for direct connection between the pulp and the periodontal ligament. Periodontal disease might trigger pulpal destruction, so, it is reasonable to anticipate that periodontitis will cause numerous teeth to lose their vitality. The link between periodontal destruction or therapy and pulpal injury has been documented [5, 6].

Interleukin-10 (IL-10) is remarkably a powerful anti-inflammatory cytokine essential for preventing tissue damage caused by excessive inflammation. IL-10 maintains immunological homeostasis by suppressing pro-inflammatory mediators and activating regulatory pathways, thereby limiting tissue destruction in inflammatory diseases [7, 8].

Nanotechnology has introduced innovative therapeutic strategies in biomedical research by enabling more efficient drug delivery mechanisms. In dentistry, nanomaterials have been explored for applications ranging from restorative materials and implants to targeted drug delivery and periodontal regeneration [9, 10]. Nanocurcumin (NCUR), a nanostructured form of curcumin, exhibits enhanced stability, solubility, and bioavailability compared with native curcumin, along with potent anti-inflammatory and antioxidant effects in experimental periodontitis models [11].

Mesenchymal stem cells (MSCs) are cells with multi potency having an innate capacity for multiple lineage transformation and self-regeneration [12, 13]. In oral tissues, bone marrow derived MSCs (BM-MSCs) can develop odontogenic lineages and contribute to the odontoblast population by production of dentin sialoprotein, highlighting their relevance for dental tissue regeneration [14].

Recent preclinical studies have increasingly focused on MSC-derived extracellular vesicles (EVs), particularly exosomes (Exo), as encouraging cell-free medicinal alternative. Exosomes are nanoscale lipid bilayer spheres (30–150 nm) which facilitate cellular connection by transmitting regulatory RNAs, proteins and lipids to recipient cells. Compared with whole cell treatments, exosomes exhibit several benefits such as higher stability, decreased immunogenicity and simpler storage requirements, making them attractive for translational regenerative medicine applications [15, 16]. Preclinical periodontitis reviews reported that MSC-derived exosome therapy significantly enhances periodontal regeneration, including improvements in bone volume and density, underscoring its therapeutic potential in inflammatory bone-loss settings [17].

Mechanistically, MSC-derived exosomes modulate cell proliferation, osteogenic differentiation, angiogenesis, and immune responses within periodontal tissues by delivering bioactive cargo, including microRNAs and growth factors. These actions facilitate tissue repair, support periodontal ligament function, and suppress chronic inflammation processes essential for effective regeneration [10, 12]. Emerging evidence further suggests that specialized exosome sources, such as those derived from neural crestrelated cell types, can enhance periodontal healing through coordinated osteogenic, angiogenic, and immunomodulatory effects, highlighting the versatility and therapeutic promise of exosome-based strategies [18, 19].

BMSC-Exo loaded by NCUR allows NCUR to be markedly bioavailable, highly soluble, and has the capacity to reach high blood concentration levels with no toxic effects. Thus, compared to other nanoparticles, exosomes may be more successful nanoparticle transporters of NCUR [20].

Despite the well-documented anti-inflammatory and regenerative properties of BM-MSC-Exo, as well as NCUR ‘s increased bioavailability, little is known about the therapeutic potential of NCUR loaded BM-MSC-Exo in preserving odontoblast integrity and modulating inflammation in periodontitis-associated conditions. The current work sought to examine the histological, histochemical, and anti-inflammatory effects of BM-MSC-Exo and NCUR loaded BM-MSC-Exo on odontoblasts in an LPS-induced periodontitis rat model.

## Materials and methods

### Experimental animals

The study used twenty-eight healthy, mature male albino rats, of age 5–7 weeks, and weight 120–150 g. The animals were acquired from Faculty of Pharmacy’s animal house at Future University in Egypt. Rats were preserved in pathogen-free polypropylene cages with 12 h light / dark cycle and monitored environmental parameters (temperature of 22 ± 2 °C, humidity ranging from 50 to 60%). Standard laboratory chow and water were afforded ad libitum right through the experimental period.

The Animal Research: Documenting of In Vivo studies (ARRIVE) rules for conducting animal research were followed in all procedures. Research Ethics Committee of Future University in Egypt granted the ethical permission (Approval No.: FUE. REC (58)/4-2026).

### Calculation of sample size

Estimation of the sample size was performed by means of MedCalc^®^ statistical software (version 12.3.0.0; MedCalc Software, Ostend, Belgium). Calculations assumed 95% confidence interval, 80% statistical power, and a 5% types I error (α). Based on effect sizes reported in previous studies [4, 19], a minimum of seven animals for each group was required, outcoming an entirely 28 rats.

### Experimental design and group allocation

After a one-week acclimatization period, rats were sorted into four groups at random by utilizing a computer-created randomized-number table (*n* = 7 per group):

Group I (Negative Control): Intra-periodontal injections of phosphate buffered saline (PBS).

Group II (Positive Control – Periodontitis): LPS injections to induce periodontitis without treatment.

Group III (Exo-treated): Periodontitis treated with BM-MSC-Exo.

Group IV (NCUR-Exo-treated): Periodontitis treated with NCUR-loaded BM-MSC-Exo.

Outcome assessors were blinded to group allocation during histological, histochemical, and biochemical analyses.

The BM-MSC-Exo and NCUR-loaded BM-MSC-Exo used in the current study were prepared using our previously published protocol [4], which included exosome isolation, TEM characterization, nanocurcumin loading, and loading efficiency experiment. As a result, the current investigation focused on assessing the biological and histopathological consequences of the previously characterized exosomes formulations.

### Induction of experimental periodontitis

Experimental periodontitis was induced in Groups II–IV by intra-periodontal injection of lipopolysaccharide (LPS) from Escherichia coli (O111:B4). A 3-µL dose of LPS solution (10 mg/mL) was injected adjacent to the bilateral lower first molars using Hamilton-type micro syringe with 30-gauge needles [21].

Injections were administered three times per week for four consecutive weeks by a trained operator to ensure reproducibility and minimize tissue trauma [22]. Group I received an equivalent volume of PBS at the same sites.

### Preparation of BM-MSC- Exo

BM-MSCs were segregated from 4-week-old rats and cultured under standard conditions. Exosomes were harvested from prepared media by Sequential ultra centrifugal technique, then purified and characterized according to our previously published protocol [4].

### Loading of nanocurcumin into exosomes

NCUR was loaded into BM-MSC-Exo via sonication. Briefly, 200 µg of NCUR was mixed with 200 µg of exosomes in PBS. The mixture underwent six cycles of sonication (30 s on/30 seconds off) for a total of 3 min per cycle, with 2-minute cooling intervals to prevent thermal degradation, using a Branson Model 3510 ultrasonic cleaner (UK). Successful loading was confirmed by UV–visible spectrophotometry according to our previously published work [4].

### Treatment protocol

After periodontitis induction, animals received a single intra-periodontal injection at the previously described sites:

Group III: 200 µg BM-MSC-Exo in PBS.

Group IV: 200 µg BM-MSC-Exo loaded with 200 µg of NCUR in PBS.

All injections were performed under aseptic conditions.

### Tissue collection and processing

Four weeks after treatment completion, animals were euthanized using cervical dislocation by dissociation anesthesia. The mandibular first molar regions were carefully dissected.

Experimental tissues were immediately cryopreserved in liquid nitrogen followed by storage in -80 °C for biochemical testing.

Mandibular first molar areas were fixed for 24 h in 4% paraformaldehyde solution at 4 °C for histological and histochemical evaluation.

### Histological and histochemical examination

Fixed specimens were decalcified, processed, followed by paraffin embedding. Serial sections of 4-µm were set for staining as follows:

Hematoxylin and Eosin (H&E): for general histologic assessment of odontoblasts and dentin.

Masson’s Trichrome Stain: to differentiate immature newly formed collagen fibers (blue) from mature collagen fibers (red).

Sections were inspected and photographed at 400× magnification utilizingmounted digital camera on top of a light microscope.

### Morphometric analysis

Quantitative assessment of newly formed collagen was accomplished by Image J software (version 1.34; NIH, USA). Seven non-overlapping fields per specimen were analyzed, and the mean area% of new collagen formation was calculated.

### Measurement of interleukin-10 (IL-10)

IL-10 amounts were evaluated by a commercial enzyme-linked immunosorbent assay (ELISA) kit (Elabscience, USA; CAT No. E-EL-R0016; identification limit 31.25–2000 pg/mL) depending on directions attained from the manufacturer.

Frozen tissues were flushed by iced PBS (0.01 M, pH 7.4), homogenized in PBS containing 0.1-1% Triton X − 100 and inhibitors of protease, then centrifugation was done for 10–15 min at 2–8 °C at 5000×g. The filtrate was gathered, and protein levels were verified by Bradford assay. Specimens were saved at -80 °C till analysis. Absorption was calculated at 450 nm with a microplate reader. Analyses were conducted at Nawah Scientific Center, Cairo, Egypt.

### Statistical analysis

The statistical software SPSS program, version 17, (IBM Corporation, Somers, New York, USA) was used to analyze the data. Quantitative variables were conveyed as mean ± standard deviation (SD). Differences among groups were analyzed by applying one-way analysis of variance (ANOVA), accompanied by The Bonferroni post hoc multiple comparisons. The data were normally distributed, Kolmogorov–Smirnov normality test was used. The standard for statistically significant results was established at *p* < 0.05.

## **Results**

### Histological results

#### Group I (Negative control)

**Fig. 1:**
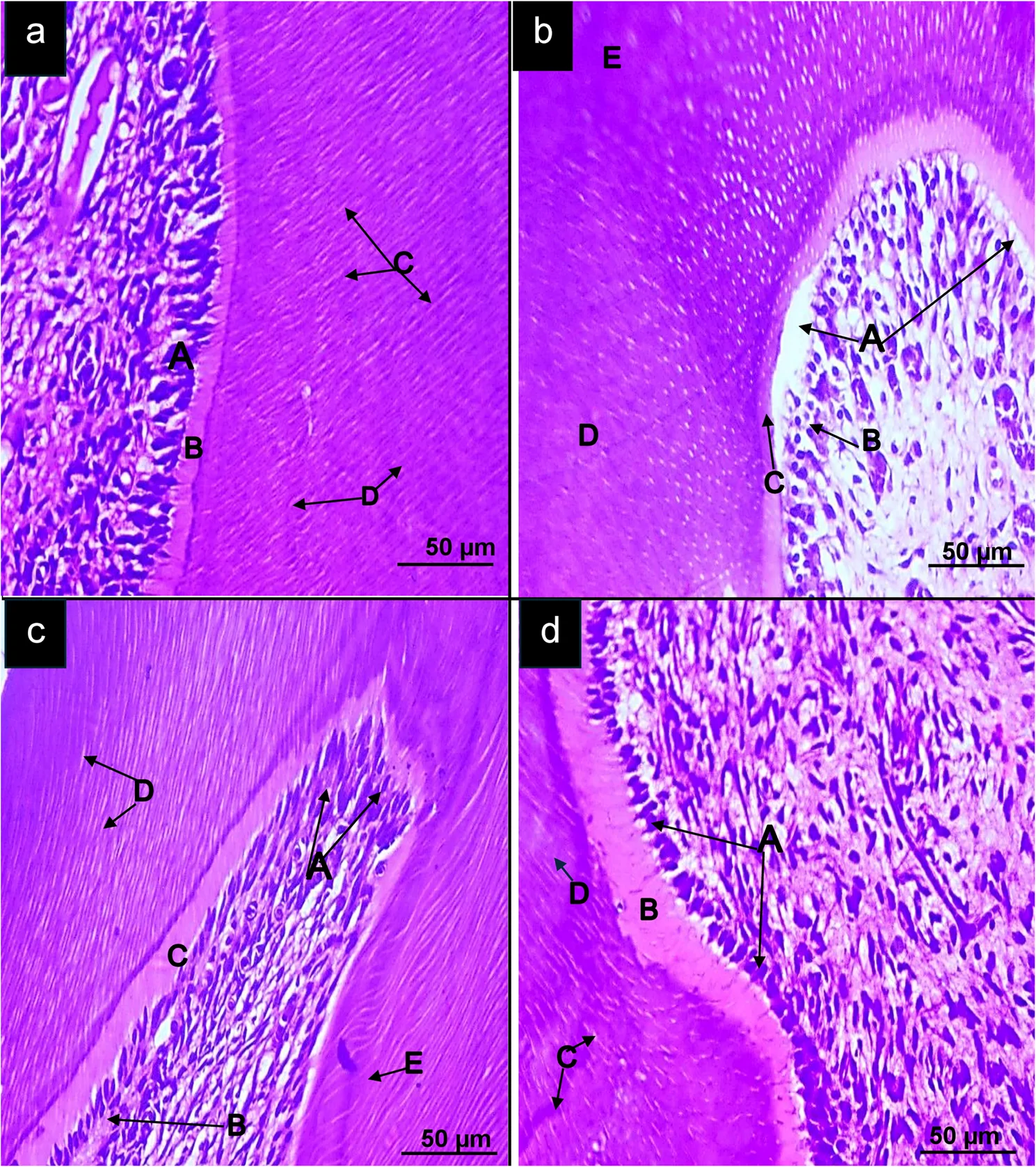
H&E-stained sections showing: **a**- Group I exhibited continuous layer of tall columnar odontoblasts (**A**). Well defined layer of predentin (**B**). Dentin with well-defined parallel dentinal tubules (**C**) and intertubular dentin (**D**). **b**- Group II displayed discontinued odontoblastic layer with some detached and desquamated odontoblasts (**A**). Most odontoblasts apparently lose their cell height and appear shrunken (**B**). Some areas showing narrow predentin (**C**). Dentin with ill-defined dentinal tubules and intertubular dentin (**D**), areas of different stainability (**E**). **c**- Group III exhibited continuously wide layer of apparently numerous odontoblast-like cells with some cells have tall columnar shape (**A**) while others are cuboidal (**B**). Well-defined continuous layer of predentin (**C**). Well defined dentinal tubules (**D**) and intertubular dentin (**E**). **d**- Group IV showing Incessant layer of tall columnar odontoblast-like cells with basal oval nuclei (**A**). Wide, continuous and distinct predentin layer (**B**). Well-demarcated parallel dentinal tubules (**C**) and intertubular dentin (**D**). (H&E x400)

Inspection of Group I histologically displayed regular dentin and dental pulp architecture. Odontoblasts were aligned as an uninterrupted, well-organized layer along the pulp boundary. Cells appeared tall and columnar, having basally situated oval nuclei, indicating proper polarization. Cytoplasmic processes extended into the dentin, reflecting normal odontoblastic activity. A clearly defined, relatively wide predentin layer was observed adjacent to the odontoblasts. Dentinal tubules were well-organized and parallel, with distinct intertubular dentin (Fig. 1a).

#### Group II (Positive control)

Sections exhibited marked disruption of odontoblastic architecture. The odontoblastic layer was discontinuous, with detachment of some cells from the dentinal surface and exfoliated into the pulp. Most odontoblasts have lost their characteristic height and appeared shrunken, vacuolated and unevenly aligned, indicative of cellular degeneration. The predentin layer appeared of uneven thickness with narrowing in some areas. Dentinal tubules and inter-tubular dentin were irregular and exhibited variable staining patterns, suggesting early structural deterioration (Fig. 1b).

#### Group III (BM-MSC-Exo treatment)

Group III demonstrated marked histological improvement compared with Group II. A broad, continuous layer of odontoblastic-like cells was observed. Certain cells retained elongated morphology having oval and basally positioned nuclei, however other cells appeared cuboidal. Cellular extensions were found to cross into the dentin. The pre-dentin layer was almost continuous and well-identified. Dentinal tubules and inter-tubular dentin were distinct, reflecting active reparative processes (Fig. 1c).

#### Group IV (NCUR-Loaded BM-MSC-Exo treatment)

Group IV exhibited nearly normal pulp-dentin architecture. A continuous and well-organized odontoblast-like cell layer was evident. Cells appeared elongated, with deeply stained oval and basally situated nuclei, and their cytoplasmic processes extended into the dentin. The predentin layer was wide, continuous, and clearly defined. Dentinal tubules and intertubular dentin closely resembled the normal structure observed in Group I (Fig. 1d).

### Masson’s trichrome staining results

**Fig. 2:**
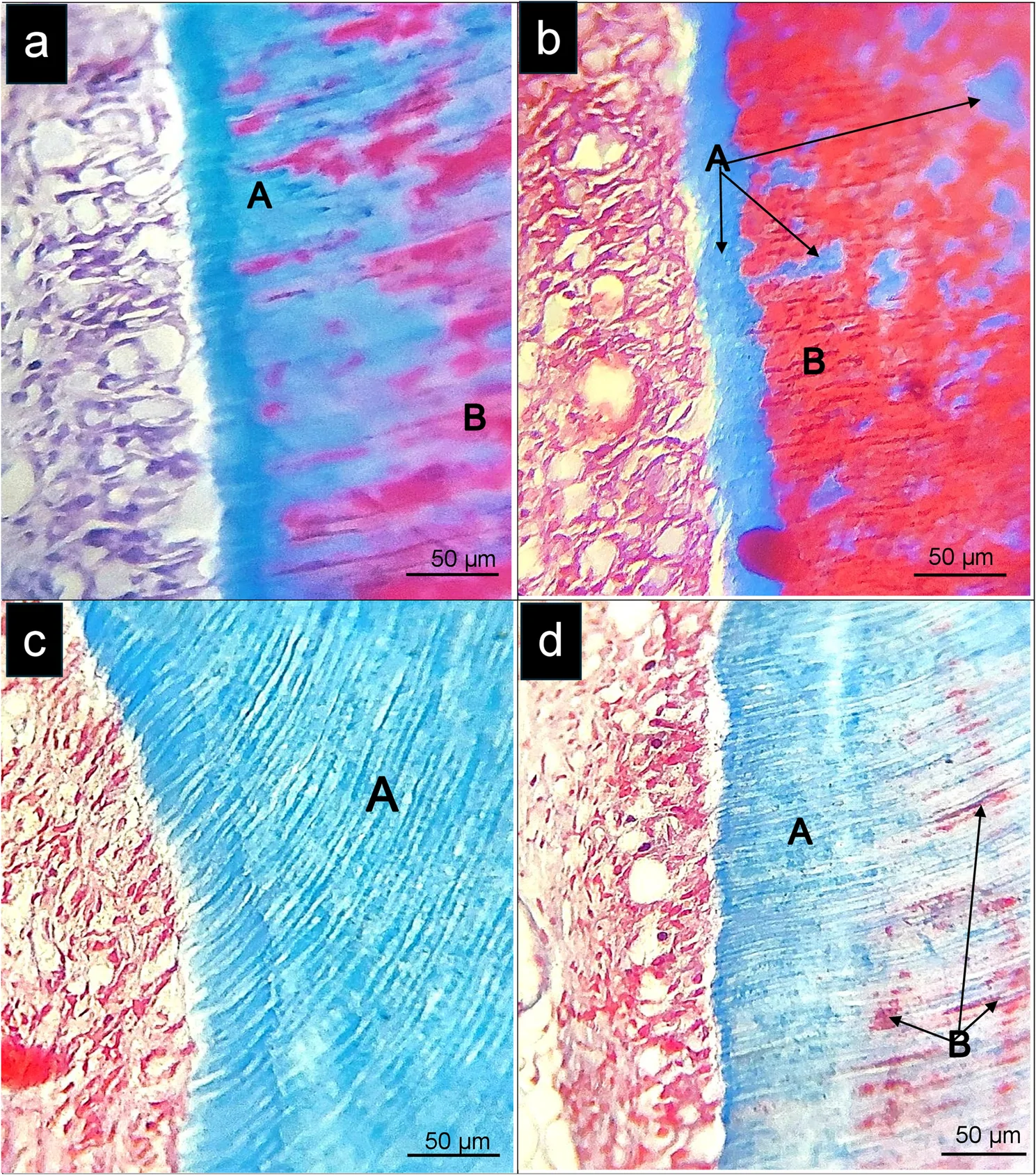
Masson’s trichrome staining sections showing **a**- Group I exhibited both newly formed (**A**) and mature collagen (**B**), indicating normal extracellular matrix maintenance and reparative activity. **b**- Group II with only sparse areas of new collagen (**A**) limited to narrow predentin regions and some peritubular dentin in a few dentinal tubules and primarily contained mature collagen (**B**). **c**- Group III displayed exclusively newly formed collagen throughout the dentin (**A**), reflecting robust regenerative activity. **d**- Group IV exhibited both newly formed (**A**) and mature collagen (**B**), indicating normal extracellular matrix maintenance and reparative activity. (Masson’s trichrome x400)

Collagen deposition in dentin was assessed using Masson’s trichrome staining. Groups I and IV exhibited both newly formed and mature collagen, indicating normal extracellular matrix maintenance and reparative activity (Fig. 2a and d). In contrast, Group II primarily contained mature collagen, with only sparse areas of newly formed collagen confined to narrow predentin regions and to some peritubular dentin in limited dentinal tubules (Fig. 2b). Remarkably, Group III displayed exclusively recent collagen formation throughout the dentin, reflecting robust regenerative activity (Fig. 2c).

### Statistical analysis

#### Newly formed collagen area (%)

**Fig. 3:**
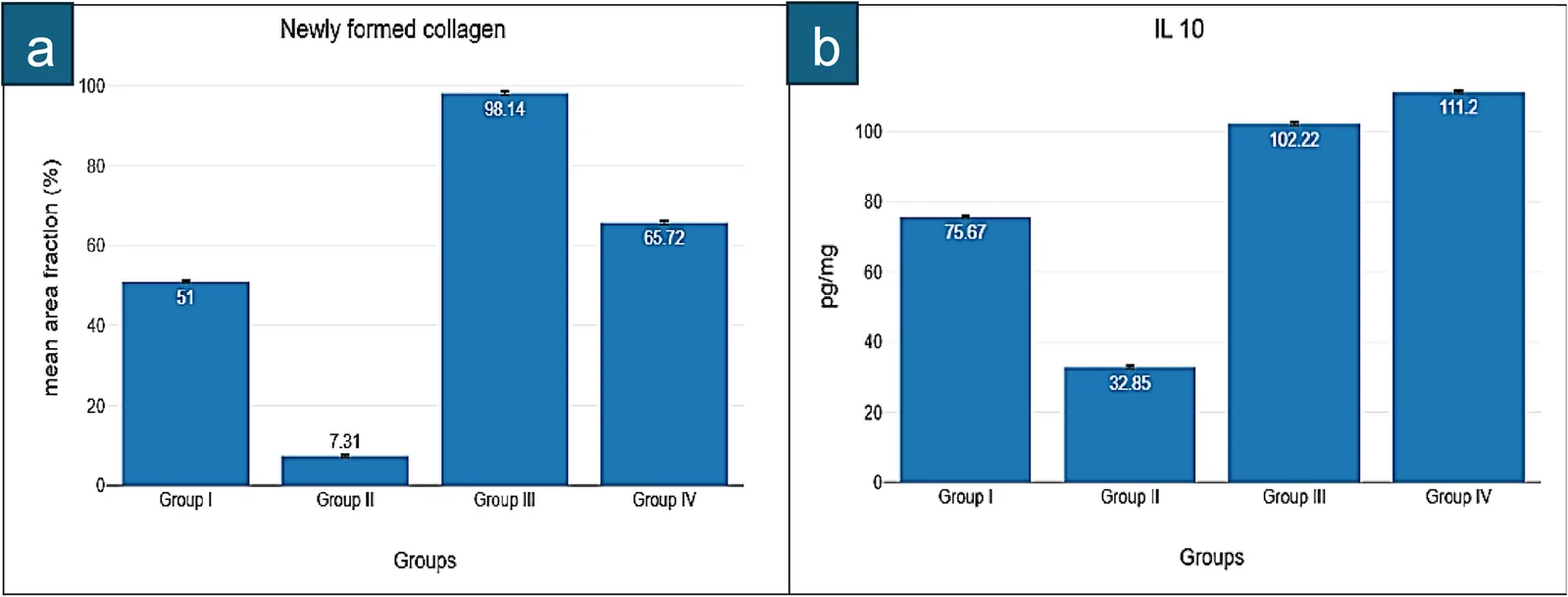
**a** Bar chart representing the mean area fraction (%) of newly formed collagen for groups, **b** Bar chart representing the mean IL-10 in all groups

Morphometric analysis of newly formed collagen revealed significant statistical variations among all experimental groups (*p* < 0.001). Group III identified the uppermost mean collagen area percentage (98.14 ± 0.49), subsequently, Groups IV (65.72 ± 0.44) and I (51 ± 0.31), while Group II showed the lowest values (7.31 ± 0.37) (Table 1; Fig. 3a).

**Table 1:** Showing the mean ± SD readings, findings of Bonferroni Post-hoc-Tests and ANOVA to compare new collagen formation mean area percentage in all experimental groups

Groups	*N*	Mean ± Std.	ANOVA (F)	*p*- value
GI	7	51 ± 0.31^bcd^	53038.38	< 0.001**
GII	7	7.31 ± 0.37 ^acd^		< 0.001**
GIII	7	98.14 ± 0.49 ^abd^		< 0.001**
GIV	7	65.72 ± 0.44^abc^		< 0.001**

Depending on *Bonferroni* post- hoc- test, superscript letters indicated the following; a: significant difference with GI, b: significant difference with GII, c: significant difference with GIII, d: significant difference with GIV

** Highly significant was considered at *p*-value < 0.001

#### Interleukin-10 (IL-10) Expression

IL-10 levels were significantly higher across all treated groups more than Group II (*p* < 0.001). Group II exhibited the lowest IL-10 levels (32.85 ± 0.43), consistent with impaired anti-inflammatory activity. IL-10 levels were highest in Group IV (111.2 ± 0.43), followed by Groups III (102.22 ± 0.47) and I (75.67 ± 0.33), reflecting enhanced anti-inflammatory responses in the treated groups (Table 2; Fig. 3b).

**Table 2:** Showing the mean ± SD readings, findings of Bonferroni Post-hoc-Tests and ANOVA to compare IL-10 values in all experimental groups

Groups	*N*	Mean ± Std.	ANOVA (F)	*p*- value
GI	7	75.67 ± 0.33^bcd^	64865.91	< 0.001**
GII	7	32.85 ± 0.43^acd^		< 0.001**
GIII	7	102.22 ± 0.47^abd^		< 0.001**
GIV	7	111.2 ± 0.43^abc^		< 0.001**

Depending on *Bonferroni* post- hoc test, superscript letters indicated the following; a: significant difference with GI, b: significant difference with GII, c: significant difference with GIII, d: significant difference with GIV

** Highly significant was considered at p-value < 0.001

## Discussion

BM-MSCs have drawn notable interest in regenerative therapy due to their pluripotent differentiation potential, self-renewal capacity, and resistance to hypoxia- and oxidative stress-induced damage. These features make BM-MSCs particularly valuable for tissue repair in chronic inflammatory conditions such as periodontitis [23].

Male Wistar albino rats were selected to eliminate potential confounding effects of female hormonal fluctuations on periodontal inflammation and repair [24]. This strain is well-established in experimental periodontitis research, providing reproducible and reliable models for studying regenerative interventions [25]. The normal histological features observed in Group I, including well-organized odontoblasts and dentin, are consistent with previous observations of healthy rat pulp tissues [26].

Exosomes have recently been recognized as a promising acellular therapeutic modality for enhancing tissue repair in periodontal diseases and bone injuries [27]. In the here in research, exosomes were isolated from BM-MSCs obtained from 4-week-old rats, consistent with prior evidence that exosomes derived from younger MSCs exhibit higher regenerative potential, including enhanced collagen production and bone apposition in fracture models [28]. The dose of 200 µgBM-MSC-Exo employed here has been validated in prior studies as optimal for therapeutic efficacy in calvaria defect repair [29]. Subgingival injection was chosen as a minimally invasive, targeted delivery route that ensures rapid local uptake while minimizing systemic exposure and treatment costs [3, 14].

The degenerative changes observed in Group II are likely attributable to the inflammatory and oxidative stress pathways activated during periodontitis. Periodontal inflammation stimulates tumor necrosis factor-alpha (TNF-α) production and enhances oxidative stress, which can inhibit genes involved in odontoblastic differentiation, for example bone morphogenetic protein-2 (BMP-2) and BMP-9 [30–32]. Concentration of nitrogen species and reactive oxygen, lipid peroxidation, and biomolecular damage further compromise tissue integrity [33].

Treatment with BM-MSC-Exo (Group III) elicited multiple reparative features, consistent with previous reports [34, 35]. The improvements likely result from both paracrine effects, including cytokine and growth factor secretion that modulates inflammation and may also contribute to differentiation of BM-MSCs into odontoblast-like cells, expressing dentin sialoprotein and promoting reparative tubular dentin formation [36–38]. BM-MSCs also exert anti-oxidative and cytoprotective effects on endogenous pulp stem cells, attenuating reactive species, downregulating pro-inflammatory cytokines, and secreting angiogenic and regenerative mediators that enhance tissue repair [39–43].

Nanotechnology has further enhanced regenerative strategies in dental medicine by improving drug delivery efficiency and bioavailability [44]. In this study, NCUR was utilized to improve solubility, bioavailability, and drug release by lowering granule size thus allowing formation of an amorphous phase of high energy [45]. Curcumin and NCUR have demonstrated efficacy in preserving alveolar bone, stimulating osteogenic differentiation of periodontal ligament stem cells, enhancing collagen synthesis, promoting fibroblast proliferation, and suppressing inflammatory infiltration in both periodontal and cutaneous wound models [21, 46–51]. These effects are largely mediated by antioxidant activity and modulation of enzymes such as glutathione peroxidase, catalase, and superoxide dismutase, which mitigate oxidative tissue damage [52, 53].

MSC-Exo have demonstrated substantial regenerative potential in periodontitis. Preclinical studies and systematic reviews indicate that exosome therapy enhances regeneration of periodontal bone and soft tissues, likely through immunomodulation, angiogenesis, and recruitment of regenerative cells [54, 55]. Exosomal cargo, including micro RNAs, contributes to osteogenic differentiation and matrix synthesis beyond paracrine signaling [56]. When combined with NCUR, exosomes can synergistically enhance anti-inflammatory and regenerative effects by enabling targeted delivery and protecting bioactive agents from rapid degradation [57], which supports the results obtained in Group IV. This combinatorial strategy represents a promising cell-free therapeutic platform for chronic inflammatory diseases, including periodontitis.

Histochemistry plays a crucial role in tissue analysis, as it enables the visualization and identification of specific chemical components using targeted staining reagents [58–60]. Histochemical analysis using Masson’s trichrome revealed that Group I maintained normal collagen dynamics, reflecting ongoing physiological dentinogenesis at an estimated secondary dentin formation rate of 0.5 μm/day [61]. Predentin and newly formed dentin are enriched in immature collagen (type III), whereas mature dentin predominantly contains type I collagen with minor contributions from type III and non-collagenous proteins [62]. The reduced newly formed collagen in Group II may reflect increased matrix metalloproteinase activity, which degrades collagen types I–III [63, 64]. In contrast, Group III showed an increased collagen deposition, attributable to BM-MSC paracrine effects and differentiation into odontoblast-like cells [65–68]. Group IV exhibited further enhancement, likely due to NCUR-mediated amplification of BM-MSC regenerative activity and reactionary dentin formation, which is rich in mature type I collagen. This suggests that Group III reflects an earlier regenerative phase, whereas Group IV represents a more advanced stage of tissue maturation and remodeling.

IL-10 expression, measured by ELISA, was significantly elevated in exosome-treated groups, with the highest levels observed in animals treated with NCUR-loaded BM-MSC-Exo. As a powerful anti-inflammatory cytokine, IL-10 could suppress massive immune responses through inhibition of pro-inflammatory cytokine formation, reduction of antigen presentation, and limitation of effector T-cell activation. Low IL-10 levels are associated with increased periodontal inflammation and bone loss [69].

BM-MSC-Exo have been shown to polarize macrophages toward an anti-inflammatory phenotype, boosting IL-10 production and reducing inflammatory tissue damage. Nanocurcumin’s anti-inflammatory and antioxidant properties, such as inhibiting NF-κB activation, modulating MAPK signaling pathways, and reducing oxidative stress, can enhance tissue protection and reduce pro-inflammatory cytokines [70–71].

The current study has various limitations that must be addressed when evaluating the results. First, odontoblast recovery was evaluated solely by histological examination and IL-10, with no molecular validation of odontoblast differentiation using particular markers such as dentin sialophosphoprotein (DSPP), dentin matrix protein-1 (DMP-1), or Nestin. Additional immunohistochemical and molecular investigations of inflammatory and regenerative signaling pathways, such as NF-κB, MAPK, and oxidative stress-related mediators, were not done, which limits the mechanistic interpretation of the reported therapeutic benefits. While the Exo and NCUR-loaded BM-MSC-Exo used in this study were prepared using the same protocol and previously characterized, including TEM and determination of NCUR-loading efficiency, future research would benefit from more comprehensive physicochemical characterization, such as nanoparticle size distribution, zeta potential, and exosomal surface marker analysis (CD9, CD63, and CD81). Finally, the study only included male rats and had a relatively short observation time, which limited the ability to examine sex-related changes and long-term treatment effectiveness. Future research that incorporates these experimental methodologies will give a more complete knowledge of the regenerative and anti-inflammatory properties of NCUR-Loaded BM-MSC-Exo, as well as aid their clinical translation.

## Conclusion

This study demonstrates that BM-MSC-Exo enhances odontoblast and dentin morphology and integrity in LPS-induced periodontitis, primarily through paracrine mechanisms that promote collagen deposition and modulate inflammatory responses. Loading exosomes with NCUR further amplifies these effects, resulting in marked restoration of odontoblast-like cells, improved predentin formation, and increased mature collagen deposition. The combined application of BM-MSC-Exo and NCUR represents a promising, minimally invasive, cell-free therapeutic strategy for periodontal tissue regeneration. Further studies are required to investigate long-term outcomes, systemic distribution, and underlying molecular mechanisms to support clinical translation. 

## Data Availability

The datasets used and/or analyzed during the current study are available from the corresponding author on reasonable request.
